# The Science of the Morning Fast

**Published:** 1897-06

**Authors:** 


### The Science of the Morning Fast.

Parental ignorance, general ignorance that there is even an alphabet of nutrition, is black with heathenism. There is not even a hazy idea that digestion is chemistry, with its exactions and its moral, mental and physical conditions, and there is a hunger of disease that food never satisfies, and that there is a starvation from over-feeding that has in its wake disease, moral and intellectual wreckage, and death.

The evil work begins in the nursery, where there is no limit to the feedings or resulting end to the woes of restlessness, fretfulness, disease and death. There are no human lives that can be made so perfectly peaceful as infantile lives, and yet if the end of the first year is reached with apparent health it will be due to an inherent strength of constitution.

The first step in every disease is the first morsel of food that fails to be fully digested. This results is an instant loss of general tone, with weak parts most affected. The vessels enlarge, with resulting strangling pressure on between structures, and in time a morbid condition called disease is reached, to be named, according to locality and character, as bronchial catarrh, asthma, rheumatism, eczema, etc. They all have a clearly ascertainable evolutionary history that does not involve a germ theory of origin or a germ treatment of relief, and it is barely possible that this is true of acute disease to a larger extant than is generally believed.

Every age has had its specifics, its special dispensations, whereby old disease-accounts under a high pressure for immediate settlement are to be abolished by miracles, or through remission rather than expiation. That will be the golden age of living in which the causes of disease will be met at their very inception by a paralyzing specific and life made continuously happy through "salvation by grace" only.—*Edward Hooker Dewey, M.D.*

---

A WOMAN living in Honolulu claims to be 124 years old—she is very deaf, can see, but indistinctly, but, take her altogether, she is said to be the Lulu of Honolulu.—*Med. Mirror.*

